# Critical Reflections on Reimbursement and Access of Advanced Therapies

**DOI:** 10.3389/fphar.2022.771966

**Published:** 2022-05-18

**Authors:** Steven Simoens, Katrien De Groote, Cornelis Boersma

**Affiliations:** ^1^ Department of Pharmaceutical and Pharmacological Sciences, KU Leuven, Leuven, Belgium; ^2^ Innosens, Brakel, Belgium; ^3^ Health Ecore, Zeist, Netherlands; ^4^ Open Universiteit, Heerlen, Netherlands; ^5^ University Medical Center Groningen, University of Groningen, Groningen, Netherlands

**Keywords:** advanced therapies, market access, reimbursement, cost-effectiveness, managed entry agreements, spread payments

## Abstract

**Background:** The health economic literature has questioned the cost-effectiveness and affordability of advanced therapies, proposed adjustments to value assessment frameworks, and discussed the use of outcome-based managed entry agreements and staggered payments in the last few years. The aim of this manuscript is to conduct a critical reflection on assessment criteria and access conditions for reimbursement of advanced therapies.

**Methods:** A narrative review of the peer-reviewed literature and grey literature was conducted in April 2021 by searching PubMed; Google Scholar; policy and legislative documents; websites of health technology assessment agencies, advanced therapy organisations, governmental advanced therapy innovation programmes, consultancy agencies; ISPOR conference abstracts and presentations.

**Results:** Based on the available evidence, this manuscript argues that: a) advanced therapies can be cost-effective at high prices set by manufacturers; b) the economic evaluation framework adopted by many payers under-values these products; c) advanced therapies can be affordable and may not require spread payments; d) outcome-based managed entry agreements are theoretically attractive, but challenging in practice; e) the cost-effectiveness of advanced therapies depends on the outcome-based managed entry agreement and payment approach; f) there is a role for multinational collaborations to manage reimbursement and access of advanced therapies.

**Conclusions:** This manuscript shows that there is no single approach to reimbursement and access of advanced therapies. Instead, we support a more tailored assessment of health economic aspects of advanced therapies, which considers the heterogeneity of these products and their target populations.

## Introduction

According to the European Medicines Agency, advanced therapies are medicines for human use that are based on genes, tissues or cells (European Commission, 2007). This product class encompasses gene therapy medicines, somatic-cell therapy medicines, tissue-engineered medicines, and advanced therapies in combination with (a) medical device(s). Advanced therapies are being developed or are used for a variety of indications (in such areas as oncology, central nervous system diseases, monogenetic diseases, infectious diseases, cardiovascular diseases, hematologic diseases, musculoskeletal and retinal diseases) and target a spectrum of disorders ranging from (ultra-)rare diseases to common diseases (Alliance for Regenerative Medicine, 2021).

Advanced therapies feature characteristics that pose challenges for market access and use. These biological medicines require a highly complex manufacturing process with stringent quality control requirements, which faces difficulties in upscaling and which is associated with substantial production and logistical costs. Also, clinical evidence at the time of launch tends to be immature and suffer from methodological limitations, and there is uncertainty about long-term health gain (or even cure). Furthermore, advanced therapies need to be administered in highly specialised treatment centres by qualified and trained health care professionals. Additionally, advanced therapies may have broader value elements which are not captured in the economic evaluation perspective adopted by many payers, may raise ethical questions, and with that political and societal concerns. Finally, payers struggle to fund significant upfront acquisition costs of advanced therapies and find it difficult to deal with uncertainties surrounding these products. Although these characteristics also apply to other innovative medicines, they are arguably present to a greater extent in advanced therapies.

In light of these characteristics, several papers have provided recommendations about how the methodology of economic evaluation needs to be adapted to fit advanced therapies (Hettle et al., 2017; Drummond et al., 2019; Jonsson et al., 2019; Aballea et al., 2020; Angelis et al., 2020; Coyle et al., 2020; Ten Ham et al., 2020). Furthermore, several health technology assessment agencies are adjusting their value assessment frameworks and decision makers in some jurisdictions are considering adapting their reimbursement pathways to reflect the characteristics of advanced therapies (Canadian Agency for Drugs and Technologies in Health, 2018). Although accounting for the characteristics of advanced therapies runs the risk that these products are treated in a different way from a health economic perspective than other medicines, there have been numerous calls from various stakeholders in multiple countries to apply specific considerations to the reimbursement and market access of advanced therapies (van Overbeeke et al., 2021). This is similar to the way that other types of innovative medicines such as (ultra-)orphan drugs (Nestler-Parr et al., 2018), antibiotics (Simoens and Spriet, 2020), or vaccines (Annemans et al., 2021) may receive special attention in value assessment and reimbursement.

An emerging and rapidly expanding health economic literature has in the last few years questioned the cost-effectiveness of advanced therapies, has warned about the affordability of advanced therapies, has issued guidance on performing economic evaluation based on immature clinical evidence, has debated adjustments to value assessment frameworks, and has proposed outcome-based managed entry agreements and staggered payment approaches for advanced therapies. Although these general claims and proposals are well founded, the aim of this manuscript is to conduct a high-level critical reflection on market access and reimbursement aspects related to advanced therapies. Specifically, this paper advocates for a more refined and granular approach which takes into account the heterogeneity of advanced therapies developed to deliver personalised medicine, and therefore calls for a tailored assessment of health economic aspects of advanced therapies.

## Methods

### Data Sources

This manuscript drew on a narrative, structured review of the peer-reviewed literature and of the grey literature. The following data sources were searched until April 2021: PubMed, Google Scholar, policy and legislative documents, websites of health technology assessment agencies (such as the English National Institute for Health and Care Excellence, the French Haute Autorité de Santé, the Canadian Agency for Drugs and Technologies in Health, the Scottish Medicines Consortium, the Agenzia Italiana del Farmaco, the US Institute for Clinical and Economic Review, the Zorginstituut Nederland), websites of advanced therapy organisations (such as the Alliance for Regenerative Medicine), websites of governmental advanced therapy innovation programmes (such as SWElife ATMP, Cell and Gene Therapy Catapult), websites of consultancy agencies (such as the Office of Health Economics and Deloitte), conference abstracts and presentations of the International Society for Pharmacoeconomics and Outcomes Research.

### Search Terms

Our search strategy sought to identify relevant material addressing the following aspects related to the reimbursement and market access of advanced therapies: clinical evidence and cost-effectiveness, affordability and spread payments, value assessment and outcome-based managed entry agreements, and multinational collaborations. Search terms related to economic evaluation (cost-effectiveness, cost-utility, cost-benefit, value, value for money, value assessment framework), market access (budget impact, affordability, pricing, reimbursement, managed entry agreement, payment), advanced therapy (cell therapy, gene therapy, trade and international non-proprietary names of specific advanced therapy products), alone and in combination with each other.

### In/Exclusion Criteria

No restrictions were placed on the type of study considered for inclusion. Aspects related to research and development, marketing authorisation, hospital exemption and business models for advanced therapies fell outside the scope of this manuscript.

There were no geographic search restrictions. Health economic evidence for all advanced therapy products (including those whose marketing authorisation has been withdrawn in specific jurisdictions) was included. Documents written in English, Dutch, French or German were considered.

### Data Analysis

All data used in this manuscript were taken from publicly available sources and references. Although this study was funded by a pharmaceutical company, the funder was not involved in the study design, collection, analysis, interpretation of data, or the writing of this article. The critical reflections formulated in the following sections represent the views of the authors and not necessarily those of the funder.

## Critical Reflections

### Advanced Therapies can be Cost-Effective at High Prices set by Manufacturers

Prices of single-dose advanced therapies have been reported to amount to up to $2 million (Angelis et al., 2020) and such high prices inhibit the cost-effectiveness of advanced therapies. Nevertheless, advanced therapies can be cost-effective if high acquisition costs are offset by sufficient health gains. For instance, a comparative analysis demonstrated that advanced therapies generate larger health gains than regular chemical and biologic medicines (Chambers et al., 2019). This particularly holds for advanced therapies which target diseases in children and young adults (e.g., Kymriah® for children and young adults with relapsed or refractory B-cell acute lymphoblastic leukemia, Zolgensma® for spinal muscular atrophy), for which advanced therapies have the potential to generate larger health gains over a patient’s lifetime.

The literature comprises economic evaluations demonstrating that some advanced therapies are cost-effective, while others are not, depending on factors such as the setting and the choice of comparator. Multiple economic evaluations have found that incremental cost-effectiveness ratios of advanced therapies exceed standard cost-effectiveness threshold values (Lin et al., 2019; Cher et al., 2020; Connock et al., 2020; Furzer et al., 2020; Viriato et al., 2020; National Institute for Health and Care Excellence, 2021a). On the other hand, there are numerous examples of advanced therapies being cost-effective from a payer perspective in general or in specific “favourable” scenarios (e.g., assumption of a lifetime horizon in line with the advanced therapy promise of a cure, application of lower discount rates for costs and outcomes). Several such examples derived from the peer-reviewed literature and from submissions to health technology assessment agencies have been listed in Table 1. To support the validity of this critical reflection, examples relate to different advanced therapies in various jurisdictions across the world.

**TABLE 1:** Examples of cost-effective advanced therapies based on economic evaluation from payer perspective.

Country	Indication	Comparator(s)	Specific design characteristics	ICER	Threshold value
Imlygic®					
England, National Institute for Health and Care Excellence, (2019c)	Unresectable, metastatic melanoma for which systemic immunotherapy is not suitable	-Dacarbazine	Lifetime horizon, 3.5% discount rate for costs and outcomes	£23,900/QALY vs. dacarbazine, £24,100/QALY *vs*. best supportive care	£20,000–£30,000/QALY
		-Best supportive care			
Kymriah®					
Japan, Wakase et al. (2021)	Children and young adults with r/r B-ALL	-Blinatumomab	Based on 5-years kymriah® trial data, lifetime horizon, 2% discount rate for costs and outcomes	¥2,035,071/QALY *vs*. blinatumomab, ¥2,644,702/QALY *vs*. clofarabine + cyclophosphamide + etoposide	¥5 million/QALY
		-Clofarabine + cyclophosphamide + etoposide			
Spain, Ribera Santasusana et al. (2020)	Children and young adults with r/r B-ALL	Salvage chemotherapy	Lifetime horizon, 3% discount rate for costs and outcomes	€28,819/QALY	€30,000/QALY
United States, Hao et al. (2017)	Children and young adults with r/r B-ALL	-Clofarabine monotherapy	20-years time horizon, 3% discount rate for costs and outcomes	Value-based price (at $100,000/QALY threshold) of Kymriah® was $505,999 *vs*. clofarabine monotherapy; $557,141 *vs*. clofarabine combination therapy; $491,442 *vs*. blinatumomab; $488,470 *vs*. other salvage chemotherapies; $663,965 *vs*. allogeneic stem cell transplant	
		-Clofarabine combination therapy			
		-Blinatumomab			
		-Other salvage chemotherapies			
		-Allogeneic stem cell transplant			
Netherlands, Thielen et al. (2020)	Children with r/r B-ALL	-Clofarabine monotherapy	Lifetime horizon, 4% discount rate for costs, 1.5% discount rate for outcomes	€27,443/QALY vs. clofarabine monotherapy; €28,611/QALY vs. clofarabine combination therapy; €23,229/QALY vs. blinatumomab	€20,000–€80,000/QALY
		-Clofarabine combination therapy			
		-Blinatumomab			
Luxturna®					
United States, Johnson et al. (2019)	RPE65-mediated inherited retinal disease	Standard care	Lifetime horizon, 3% discount rate for costs and outcomes	$79,618/QALY	$50,000-$100,000/QALY
United States, Zimmermann et al. (2019)	RPE65-mediated inherited retinal disease	Standard care	Assuming immediate restoration to normal vision during remaining lifetime, 3% discount rate for costs and outcomes	$52,000/QALY	$50,000–$100,000/QALY
Spherox®					
England, Armoiry et al. (2019)	Knee articular cartilage defects	-Microfracture	Lifetime horizon, 3.5% discount rate for costs and outcomes	£4,360/QALY *vs*. microfracture, around £18,000/QALY *vs*. MACI	£20,000–£30,000/QALY
		-MACI			
Strimvelis®					
England, South et al. (2019)	Adenosine deaminase deficiency–severe combined immunodeficiency	Haematopoietic stem cell transplant from haploidentical donor	Lifetime horizon, 1.5% discount rate for costs and outcomes	£16,704/QALY	£20,000–£30,000/QALY
Valoctocogene roxaparvovec					
United States, Cook et al. (2020)	Severe haemophilia A	Prophylactic FVIII replacement therapy	Assuming price similar to that of currently available gene therapies, lifetime horizon, 3% discount rate for costs and outcomes	Dominant	$50,000–$100,000/QALY
Yescarta®					
Italy, Marchetti et al. (2018)	Adults with r/r DLBCL	Salvage chemotherapy	Lifetime horizon, 3% discount rate for costs and outcomes	€44,746/QALY	€50,000/QALY
United States, Roth et al. (2018)	Adults with r/r DLBCL	Salvage chemotherapy	Lifetime horizon, 3% discount rate for costs and outcomes	$58,146/QALY	$50,000-$100,000/QALY
United States, Whittington et al. (2019)	Adults with r/r DLBCL	Salvage chemotherapy	Lifetime horizon, 3% discount rate for costs and outcomes	$82,400/QALY	$50,000-$100,000/QALY
Zolgensma®					
United States, Malone et al. (2019)	Spinal muscular atrophy 1 patients with 2 copies of SMN2 gene	Nusinersen	Lifetime horizon, 3% discount rate for costs and outcomes	Dominant (at drug price of $4 million)–$31,379/QALY (at drug price of $5 million)	$50,000-$100,000/QALY
Zynteglo®					
France, Autorité de Santé, (2020)	Transfusion-dependent β-tha-lassaemia	Red blood cell transfusions and iron chelation	Lifetime horizon, 0% discount rate for costs and outcomes	Dominant	-
Sweden, FiNoSe, (2019)	Transfusion-dependent β-tha-lassaemia	Red blood cell transfusions and iron chelation	Lifetime horizon, 3% discount rate for costs and outcomes	583,767 SEK/QALY (at 0% discount rate for outcomes)	700,000–1,220,000 SEK/QALY

Notes: *Formal threshold value or threshold value commonly proposed in literature for a country.

ICER, incremental cost-effectiveness ratio; MACI, matrix-applied characterised autologous cultured chondrocyte implant; QALY, quality-adjusted life year; r/r B-ALL, relapsed/refractory B-cell acute lymphoblastic leukemia; r/r DLBCL, relapsed/refractory diffuse large B-cell lymphoma.

It has to be acknowledged that sensitivity analyses of these economic evaluations consistently show that the advanced therapy price is a major driver of cost-effectiveness. In this respect, many economic evaluations draw on publicly available list prices in their analysis, even though price discounting is common for many types of medicines including advanced therapies. Hence, these studies do not account for discounts following negotiations between manufacturers and payers, which improve cost-effectiveness. Also, some payers revise cost-effectiveness estimates based on net prices with a view to inform their assessment, even though such results are typically not publicly disclosed. Finally, one United States economic evaluation calculated the value-based price at which Kymriah® would be cost-effective using a $100,000 per quality-adjusted life year gained threshold and found that these prices (which depended on the choice of comparator) surpassed the actual Kymriah® price of $475,000 (Hao et al., 2017).

How does the cost-effectiveness of advanced therapies compare to that of alternative treatments for the same diseases? A study compared the cost-effectiveness of CAR-T therapies (calculated by the United States Institute for Clinical and Economic Review in 2018) with the cost-effectiveness of other medicines and of non-pharmaceutical treatments for cancer (as derived from US cost-utility analyses included in the Cost–Effectiveness Analysis Registry of the Tufts Medical Center) (Baumgardner et al., 2020). The results indicated that there was no statistically significant difference in cost-effectiveness between CAR-T therapies and other medicines, and between CAR-T therapies and non-pharmaceutical treatments.

### The Economic Evaluation Framework Adopted by Many Payers Under-Values Advanced Therapies

An economic evaluation from a payer perspective does not capture the full cost-effectiveness given that it does not consider the impact on patient education and productivity, and on informal caregiver productivity and health. Although this is the case for all types of medicines, it is particularly relevant for advanced therapies and these products may be associated with additional value elements such as the value of cure, the value of scientific spill-overs [for instance, the COVID-19 vaccines developed by Moderna and by Pfizer/BioNTech built on their expertise regarding advanced therapies (American Society of Gene and Cell Therapy, 2020)], real-option value and insurance value (Lakdawalla et al., 2018). To the best of our knowledge, no economic evaluation of an advanced therapy has included such additional value elements to date.

Multiple research teams have explored changes to the methodology of economic evaluation to account for the characteristics of advanced therapies (Hettle et al., 2017; Drummond et al., 2019; Jonsson et al., 2019; Aballea et al., 2020; Angelis et al., 2020; Coyle et al., 2020; Ten Ham et al., 2020). These papers focus on issues such as the choice of a payer or societal perspective, the heterogeneity of target population and of treatment effect, the durability of health gain and data extrapolation techniques, the availability of a single-arm advanced therapy trial and comparison with a historical cohort, the validation of surrogate outcomes, the application of lower or different discount rates for costs and outcomes, the consideration of disease severity, and the inclusion of broader value elements.

Although a discussion of these methodological issues falls outside the scope of this manuscript, choices on how to address these issues when conducting an economic evaluation influence the cost-effectiveness of advanced therapies. Here, we provide three examples of economic evaluations of advanced therapies which were included in Table 1, and report how the cost-effectiveness of these products changes when a broader societal perspective is taken instead of a payer perspective:

• Treatment of Japanese children and young adults with relapsed/refractory B-cell acute lymphoblastic leukemia with Kymriah® was cost-effective as compared to blinatumomab and as compared to clofarabine + cyclophosphamide + etoposide from a payer perspective. Kymriah® was more effective and less expensive than either comparator when costs of productivity loss were also considered in the economic evaluation from a societal perspective (Wakase et al., 2021).
• A Dutch economic evaluation of Kymriah® for children with relapsed/refractory B-cell acute lymphoblastic leukemia included travel costs, costs of caregiver productivity loss and hotel stay, and costs of informal care in the analysis from a societal perspective (Thielen et al., 2020). Incremental costs per quality-adjusted life year gained of Kymriah® were higher from the societal than from a payer perspective, but Kymriah® remained cost-effective.
• An economic evaluation calculated the cost-effectiveness of Luxturna® for *RPE65*-mediated inherited retinal disease in the United States (Johnson et al., 2019). This product was cost-effective from a payer perspective, and became more effective and less expensive than standard care from a societal perspective (which also accounted for educational costs, costs of productivity loss, caregiver burden, and costs of government programmes for people with visual impairment).

The methodological discussion is also mirrored in guidance about the economic evaluation of advanced therapies issued by regulatory agencies. For instance, the 2021–2024 agreement between the French Economic Committee for Health Products (*CEPS, le Comité Economique des Produits de Santé*) and the Pharmaceutical Industry Association (*LEEM, les Entreprises du Médicament*) contains advanced therapy-specific stipulations regarding comparator, uncertainties, price discounts, spread payments and contracts related to real-life transferability (CEPS and LEEM, 2021). In the United States, the Institute for Clinical and Economic Review has changed its economic evaluation methodology with respect to addressing uncertainties associated with advanced therapies, considering value of cure and real-option value, and sharing health care cost offsets (Institute for Clinical and Economic Review, 2019a).

Therefore, this manuscript calls for evolution rather than revolution when it comes to economic evaluation of advanced therapies: these products do not require a radically new value assessment framework, but payers could consider a broader set of advanced therapy characteristics and value elements in addition to those from their traditional perspective. Indeed, our call to take a societal perspective in value assessment is not limited to advanced therapies, but needs to be extended to all types of medicines. As a result, some health technology assessment agencies are adjusting their value assessment frameworks (see Table 2). For instance, Canada has implemented a distinct review process for cell and gene therapies that integrates evidence requirements from the medicines and medical devices review processes (Canadian Agency for Drugs and Technologies in Health, 2018). Multiple European jurisdictions have in place adapted value assessment frameworks that account for disease severity or that are specific to (ultra-)orphan medicines, or medicines with a high incremental cost per quality-adjusted life year gained (Scottish Medicines Consortium, 2012; Svensson et al., 2015; Zwaap et al., 2015; WHO Collaborating Centre for Pharmaceutical Pricing and Reimbursement Policies, 2018; Healthcare Improvement Scotland, 2019; National Institute for Health and Care Excellence, 2022). These frameworks are likely to be applicable to advanced therapies to the extent that such products fall within their scope. For example, the National Institute for Health and Care Excellence in England considered Tecartus® to be a life-extending treatment at the end of life (National Institute for Health and Care Excellence, 2021b) and assessed Luxturna® in its Highly Specialized Technologies programme (National Institute for Health and Care Excellence, 2019d).

**TABLE 2:** Examples of adapted value assessment frameworks that apply to (some) advanced therapies.

Country	Value Assessment Framework	
	Scope	Description
Belgium	Orphan medicines	Orphan medicines are classified as medicines with added therapeutic value and exempted from requirement to conduct economic evaluation
Canada, Canadian Agency for Drugs and Technologies in Health, (2018)	Cell and gene therapies	Consideration of multiple elements, including efficacy, safety, cost-effectiveness, budget impact, patient and clinician engagement, ethical and implementation issues
England, National Institute for Health and Care Excellence, (2022)	Health technologies	Weighted QALYs for severe diseases depending on absolute and proportional shortfall in QALYs
Netherlands, Zwaap et al. (2015)	Health technologies	Variable threshold value (€20,000-€50,000-€80,000/QALY) depending on disease severity
Norway, WHO Collaborating Centre for Pharmaceutical Pricing and Reimbursement Policies, (2018)	Health technologies	Higher threshold value for severe diseases
Scotland	Medicines, Scottish Medicines Consortium, (2012)	If high cost/QALY, consideration of additional elements, including substantial improvement in health, specific health gain in patient sub-group, absence of therapeutic alternatives, bridging to other proven therapy
	Ultra-orphan medicines, Healthcare Improvement Scotland, (2019)	-Consideration of multiple elements, such as disease severity, efficacy, safety, cost-effectiveness (including societal perspective), carer quality of life, staffing and infrastructure issues
		-Patient access scheme
		-Data collection arrangement
Sweden, Svensson et al. (2015)	Medicines, medical devices, dental procedures	Higher threshold value for severe diseases

Note: QALY, quality-adjusted life year.

### Economic Evaluation of Advanced Therapies Relies on Immature Clinical Evidence

Clinical evidence supporting the safety and efficacy of advanced therapies generally does not meet economic evaluation requirements in terms of study design (e.g. open-label, single-arm trial), sample size, heterogeneous patient population, surrogate outcome measures, and duration of patient follow-up (Qiu et al., 2020). This is to be expected for medicines which may target rare diseases without alternative treatments or which may have been authorised under early access or accelerated assessment pathways (van Overbeeke et al., 2021). For instance, a review of economic evaluations pertaining to six advanced therapies submitted to five health technology assessment agencies highlighted concerns about the (duration of) efficacy, the association between surrogate and final outcomes, the lack of comparative and sub-group data (Faulkner et al., 2019).

This makes it more difficult to appraise the value of advanced therapies given that the cost-effectiveness of these products tends to be sensitive to parameters such as the time horizon, assumptions regarding the duration of health gain generated by the advanced therapy, the number of years of patient follow-up in single-arm trials and survival extrapolation techniques (Hao et al., 2017; Marchetti et al., 2018; Roth et al., 2018; Armoiry et al., 2019; National Institute for Health and Care Excellence, 2019c; FiNoSe, 2019; Johnson et al., 2019; Malone et al., 2019; South et al., 2019; Walton et al., 2019; Whittington et al., 2019; Zimmermann et al., 2019; Autorité de Santé, 2020; Cook et al., 2020; Ribera Santasusana et al., 2020; Thielen et al., 2020; Wakase et al., 2021). In light of such clinical uncertainties, the French High Council for Health (*HAS, Haute Autorité de Santé*) for example has demanded recurrent annual economic evaluations of Kymriah® and Yescarta® incorporating the most recent data (Jorgensen and Kefalas, 2021).

Hence, there is a need for methodological guidelines on how the limitations of clinical evidence about advanced therapies can be addressed for the purpose of economic evaluation and reimbursement. For instance, an International Society for Pharmacoeconomics and Outcomes Research task force has issued guidance on how to conduct an indirect treatment comparison (when evidence from a randomised controlled trial comparing the therapies of interest is not available) or a network meta-analysis (even though multiple clinical studies may not be available for advanced therapies) (Jansen et al., 2011).

Although it is difficult to design and conduct high-quality trials for advanced therapies, practice also shows that this is not an insurmountable hurdle. For instance, marketing authorisation of Provenge® was based on the multi-centre, randomised, double-blinded, placebo-controlled, phase III IMPACT trial which examined overall survival in 512 men with metastatic androgen-independent prostatic adenocarcinoma over a period of 5 years (ClinicalTrials.gov, 2010). Furthermore, this manuscript argues that the bar regarding the quality of clinical evidence is likely to be raised by health technology assessment agencies in the future as more and more advanced therapies request reimbursement.

In response to immature clinical evidence about advanced therapies at the time of initial reimbursement, there is a need to collect real-world data on outcomes in routine clinical practice. Such data can serve to re-assess the cost-effectiveness of advanced therapies over time and can be generated in the context of outcome-based managed entry agreements (cfr. infra). For instance, a project is being piloted to create a European advanced therapy registry with data on patient outcomes for a particular rare disease (Horgan et al., 2020). In the future, the creation of the European Health Data & Evidence Network can also support the collection and analysis of real-world data (EHDEN, 2022).

### Advanced Therapies can be Affordable and may not Require Spread Payments

The high number of advanced therapies in development (with 1,220 ongoing clinical trials in the world in 2020) (Alliance for Regenerative Medicine, 2021) and their elevated price tag has raised concerns about their affordability among payers. As a result, some have argued that advanced therapies are ‘cost-effective, but unaffordable’ (Leech and Dusetzina, 2019).

This manuscript argues that the budget impact of advanced therapies needs to be put into perspective. Although advanced therapies are very expensive, their curative potential implies that one-off costs of advanced therapies need to be compared with lifetime costs of chronic treatments. For instance, when a study identified the top 20 most expensive orphan medicines in terms of annual treatment costs, six were advanced therapies and 14 were chronic therapies (Foxon et al., 2019). When calculating lifelong costs, the study showed that mean wholesale acquisition costs of chronic therapies (US$ 9.3 million) exceeded mean costs of advanced therapies (US$ 0.9 million). However, the authors did not account for the fact that these medicines were used to treat different diseases.

Hence, a more informative analysis contrasts the costs of an advanced therapy with lifetime costs of current chronic therapy for the disease as illustrated by the following examples. In haemophilia A, a disease for which advanced therapies are being developed, lifelong costs of prophylactic or periodic factor administration amount to US$ 5–10 million in the United States (Orkin and Reilly, 2016). Costs of heart transplantation, the only curative therapy currently available for end-stage heart failure, are US$ 1.7 million (Ali, 2020). Costs in transfusion-dependent β-thalassaemia amount to 17 million SEK with Zynteglo® and 7.2 million SEK with lifelong red blood cell transfusions and iron chelation therapy (using a 3% discount rate) in Sweden (FiNoSe, 2019). However, it needs to be recognised that advanced therapy costs are incurred at a single time point, whereas costs of chronic treatments are spread over a patient’s lifetime.

This would suggest that the budget impact associated with reimbursing advanced therapies primarily raises the problem of having to make budgetary adjustments and pay large sums at once rather than an affordability issue. Thus, if a mechanism can be set up which enables payment for advanced therapies in installments over time, this would mimic the current payment approach for chronic treatments and avoid the challenge of high one-off costs associated with advanced therapies. However, for such a mechanism to be implemented in Europe, it needs to comply with the European System of National and Regional Accounts rules (Eurostat, 2013), which state that spread payments are labelled as debt and are aggregated in a single amount that is incurred at the time of therapy administration from an accounting perspective; and with local regulations (for example, the Swedish Local Government Act restricts spreading payments to a period of 3 years (Ridderstad Wollberg, 2020)). Another theoretical solution is to consider advanced therapies as intangible assets that generate health gain and produce economic benefit (e.g., productivity gain), thus making it possible to amortise advanced therapies and spread payments over time in the payer’s income statement (Dabbous et al., 2021).

It can be questioned whether spread payment approaches are needed for all advanced therapies. Given that this is a heterogeneous class of products, such an approach may not be required for ultra-orphan advanced therapies or for advanced therapies at the lower price end. For some therapies, it appears to be a cosmetic solution allowing for a phasing towards a rather steady and thus predictable annual budget impact. Therefore, there is a need to identify disease and advanced therapy characteristics—such as disease incidence and prevalence—on the basis of which a payment approach (single payment or spread payments) for a specific advanced therapy can be chosen (Van Dyck et al., 2022).

With a view to address affordability of advanced therapies, approaches other than spread payments have been proposed in the literature. These include intellectual property-based payments, pooling pharmaceutical and health care budgets, an insulated advanced therapy fund, additional private health insurance, payer loans or re-insurance schemes, outcome-based managed entry agreements (cfr. infra), and specific combinations of these. Interviews with European and Belgian payers, however, indicated that they have little interest in these payment approaches, with the exception of outcome-based managed entry agreements with (out) spread payments (Schaffer et al., 2018; Maes et al., 2019).

At a more general level, how can we reconcile cost-effectiveness with affordability in order to support sustainable reimbursement for advanced therapies? One approach proposed in the literature is to adjust the cost-effectiveness threshold value (which is used to determine whether an advanced therapy is cost-effective) in function of the budget impact (and other relevant criteria) (Towse and Mauskopf, 2018). The rationale for this is that society’s maximum willingness to pay per unit of health gain should be higher for advanced therapies with a lower budget impact and targeting a more severe disease (Annemans, 2019). Conversely, the appraisal of an advanced therapy with a high budget impact for a less severe disease should be subjected to a lower cost-effectiveness threshold value. Although this is a promising approach, much conceptual, practical and validation research is still needed.

In practice, some health care systems and health technology assessment agencies have implemented different approaches to integrate cost-effectiveness and affordability in their value assessment framework. Here, we provide examples relating to the Dutch ‘lock for expensive medicines’, the US Institute for Clinical and Economic Review value framework, the English budget impact test, and the Italian funds for innovative medicines:

• In the Netherlands, Libmeldy®, Tecartus®, Zolgensma® and Zynteglo® have been placed in the “lock for expensive medicines” (Zorginstituut Nederland, 2021). This means that reimbursement is postponed for a period of time during which the medicine is assessed (in terms of necessity, effectiveness, cost-effectiveness and feasibility), conditions for appropriate use are agreed with health care professionals; and the Minister of Health can enter into price negotiations with the manufacturer.
• In the United States, the Institute for Clinical and Economic Review considers budget impact in its value assessment, computes a health technology’s price at which it meets particular cost-effectiveness threshold values, and performs potential budget impact analyses calculating the price and uptake levels at which a health technology reaches a specific affordability threshold (Pearson, 2018). This approach was applied to, for example, Zolgensma® (Institute for Clinical and Economic Review, 2019b).
• Since April 2017, the National Institute for Health and Care Excellence and the National Health Service England calculate the budget impact of all new health technologies (National Institute for Health and Care Excellence, 2017). If the budget exceeds £20 million in any of the first 3 years, the National Health Service England may negotiate a price discount with the manufacturer taking into account the technology’s cost-effectiveness and budget impact.
• Italy has allocated €500 million to each of two insulated funds with a view to support access to innovative medicines, with one fund dedicated to oncology medicines and the other fund comprising other medicines (Flume et al., 2018). If the budget is surpassed, the excess is paid back by pharmaceutical industry. Advanced therapies such as Kymriah®, Luxturna®, Onpattro®, Yescarta® and Zolgensma® are paid for through these funds.

### Outcome-Based Managed Entry Agreements for Advanced Therapies Are Theoretically Attractive, but Challenging in Practice

Market access of advanced therapies is associated with clinical uncertainty given that follow-up data on clinical efficacy tend to be short term at the time of reimbursement application. To address this clinical uncertainty, outcome-based managed entry agreements have been proposed for advanced therapies in the literature. Such agreements link payment to the product’s observed efficacy in real-life clinical practice (Dabbous et al., 2020). Hence, payers are considering and implementing outcome-based managed entry agreements (alone or in combination with financial-based managed entry agreements, with or without spread payments) for advanced therapies (see Table 3). This table also illustrates the variety of agreement types that are used for advanced therapies and shows that jurisdictions may apply different outcome-based agreements to the same advanced therapy.

**TABLE 3:** Examples of outcome-based managed entry agreements for advanced therapies.

Country	Indication	MEA Type	Outcome Measurement	Payment Schedule
Holoclar®				
Italy, McConaghie, (2019)	Limbal stem-cell deficiency due to burns to the eyes	Pay-for-performance with money-back guarantee	Absence of effectiveness at 1 year	Not reported
Kymriah®				
Belgium, National Institute for Health and Disability Insurance, (2019)	Adults with r/r DLBCL	Pay-for-performance with spread payments	Treatment response and patient condition at 6, 12, 18, and 20 months	At administration (€296,800), 12 months (€21,200), 20 months (€21,200)
	Children and young adults with r/r B-ALL	Pay-for-performance with spread payments	Treatment response and patient condition at 6 and 12 months	At administration (€296,800), 6 months (€21,200), 12 months (€21,200)
England, National Institute for Health and Care Excellence, (2019b)	Adults with r/r DLBCL	Coverage with evidence development and rebate	Overall and progression-free survival, intravenous immunoglobulin use	Confidential
France, Jorgensen and Kefalas, (2021)	Adults with r/r DLBCL	Coverage with evidence development	Survival and disease progression at 28 and 100 days, at 6 months and each following 6 months	Not reported
Germany, GWQ Service Plus, (2019)	Adults with r/r DLBCL, children and young adults with r/r B-ALL	Pay-for-performance with partial refund	Death during specific time period after administration	€320,000, size of refund not reported
Italy, Partners4Access, (2019)	Adults with r/r DLBCL, children and young adults with r/r B-ALL	Pay-for-performance with spread payments	Disease progression and death at 45 days, 6 and 12 months	At 45 days (€64,000), 6 months (€128,000), 12 months (€128,000)
Spain, Rosa, (2019); Jorgensen et al. (2020); Sheppard et al. (2021)	Adults with r/r DLBCL, children and young adults with r/r B-ALL	Pay-for-performance with spread payments	Complete treatment response at 18 months	At administration (€166,400), 18 months (€153,600)
Luxturna®				
United States, LaMattina, (2019)	*RPE65*-mediated inherited retinal disease	Pay-for-performance with rebate	Light sensitivity testing scores at 30–90 days and at 30 months	Size of rebate not reported
Strimvelis®				
Italy, Colasante, (2019)	Adenosine deaminase deficiency–severe combined immunodeficiency	Pay-for-performance with partial refund	Not reported	Staggered payments
Tecartus®				
England, National Institute for Health and Care Excellence, (2021a)	Adults with r/r MCL	Coverage with evidence development and rebate	Overall and progression-free survival	Confidential
*Yescarta®*				
Belgium, National Institute for Health and Disability Insurance, (2019)	Adults with r/r DLBCL	Pay-for-performance with spread payments	Treatment response and patient condition at 6, 12, 18 and 20 months	At administration (€304,220), 12 months (€21,200), 20 months (€21,200)
England, National Institute for Health and Care Excellence, (2019a)	Adults with r/r DLBCL or PMLBCL	Coverage with evidence development and rebate	Overall and progression-free survival, intravenous immunoglobulin use	Confidential
Germany, APM Health Europe, (2019)	Adults with r/r DLBCL or PMLBCL	Pay-for-performance with rebate	Patient survival	Not reported
Spain, Sheppard et al. (2021)	PMBCL	Pay-for-performance with spread payments	Overall survival at 18 months	At administration (€118,000), 18 months (€209,000)
Zolgensma®				
Germany, GWQ Service Plus, (2020)	Spinal muscular atrophy 1 patients	Pay-for-performance with staggered money-back guarantee	Two patient-relevant outcome measures	Not reported
United States, Remap Consulting, (2019)	Spinal muscular atrophy 1 patients	Pay-for-performance with spread payments	Not reported	Spread payments over 5 years
Zynteglo®				
Germany, Parsons, (2020)	Transfusion-dependent β-thalassaemia	Pay-for-performance with spread payments	Absence of red blood cell transfusions	At administration and 4 annual payments
Sweden, FiNoSe, (2019)	Transfusion-dependent β-thalassaemia	Pay-for-performance with spread payments	Not reported	Five payments over 4 years

Notes: MEA, managed entry agreement; PMLBCL, primary mediastinal large B-cell lymphoma; r/r B-ALL, relapsed/refractory B-cell acute lymphoblastic leukemia; r/r DLBCL, relapsed/refractory diffuse large B-cell lymphoma; r/r MCL, relapsed/refractory mantle cell lymphoma.

Although outcome-based managed entry agreements for advanced therapies are theoretically attractive, their practical design and implementation proves challenging. Indeed, a systematic literature review of barriers associated with outcome-based agreements pointed to difficulties in selecting suitable outcome measures and adjusting payment to observed outcomes; the administrative burden, personnel and infrastructure requirements for data collection; and the lack of an appropriate multi-stakeholder governance structure (Michelsen et al., 2020).

Therefore, there is a need for a legislative framework and a roadmap that provides guidance to manufacturers, payers and health care providers on how to design and implement outcome-based managed entry agreements for advanced therapies in terms of data collection, quality and analysis; outcome selection and payment correction; funding and data ownership. Several European jurisdictions are taking steps forward in this respect as exemplified by the following illustrations:

• The Spanish National Service introduced Valtermed in 2019, a web-based information system which serves to elicit the therapeutic benefit in real-life clinical practice of medicines with a significant health and economic impact (Ministerio de Sanidad, 2019). Valtermed was piloted using the cases of Kymriah® and Yescarta® (Jorgensen et al., 2020), and is proposed to be used for Luxturna® (Ministerio de Sanidad, 2020).
• The Italian Medicines Agency (*AIFA, Agenzia Italiana del Farmaco*) uses a web-based platform of monitoring registries which serve to follow up the use and therapeutic benefit of medicines in real-life clinical practice and the application of outcome-based (and financial-based) managed entry agreements (Agenzia Italiana del Farmaco, 2021). With respect to advanced therapies, such registries have been set up in the context of outcome-based agreements for Kymriah® and Yescarta® and to monitor appropriate prescribing of Onpattro®, Luxturna® and Zolgensma®.
• Since July 2016, the National Health Service Cancer Drugs Fund in England can grant conditional access to cancer medicines which are associated with clinical and cost-effectiveness uncertainty subject to the requirement that real-life data are collected during 2 years (NHS England, 2021). Data are gathered via the Systematic Anti-Cancer Therapy Dataset. Such outcome-based managed entry agreements have been set up for, for example, Kymriah® (National Institute for Health and Care Excellence, 2019b), Tecartus® (National Institute for Health and Care Excellence, 2021a) and Yescarta® (National Institute for Health and Care Excellence, 2019a), with the agreement stipulating patient eligibility, areas of clinical uncertainty, data sources, outcome measures, data analysis, data ownership and protection.

### The Cost-Effectiveness of Advanced Therapies Depends on the Accompanying Outcome-Based Managed Entry Agreement and/or the Payment Approach

Traditionally, economic evaluation calculates the cost-effectiveness of a medicine for which a payer incurs one-time payments for all target patients irrespective of actual outcome. The application to advanced therapies of managed entry agreements that tie (staggered) payment(s) to actual outcomes influences whether, when and how much of the acquisition price needs to be paid, thereby affecting the cost-effectiveness of advanced therapies from a payer perspective.

Few economic evaluations of advanced therapies from a payer perspective are publicly available that account for the specific characteristics of the accompanying outcome-based managed entry agreement and/or staggered payment approach:

• Three US economic evaluations found Kymriah® for children and young adults with relapsed/refractory B-cell acute lymphoblastic leukemia to be cost-effective in the reference case which limited payment to treatment responders (Lin et al., 2018; Whittington et al., 2018; Sarkar et al., 2019).
• When comparing Kymriah® and Yescarta® separately with salvage chemotherapy and stem-cell transplantation for adults with relapsed/refractory diffuse large B-cell lymphoma, a United States economic evaluation showed that cost-effectiveness improved when an outcome-based agreement was implemented which restricted payment to patients with complete/partial response (Lin et al., 2019).
• A French economic evaluation explored the sensitivity of the incremental cost-utility ratio of Zynteglo® as compared with red blood cell transfusions and iron chelation to changes in the payment approach (Autorité de Santé, 2020). The ratio amounted to €151,003 per quality-adjusted life year gained under a one-time payment and improved to €106,307 per quality-adjusted life year gained when annual payments were spread over 5 years (and were discounted to calculate their present value).

### There is a Role for Collaborations Between Countries to Manage Market Access and Reimbursement of Advanced Therapies

Advanced therapies are complex products, often registered for rare diseases or targeted treatment for severe diseases, and their market access and reimbursement tends to involve the conduct of methodologically challenging economic evaluations, the implementation of sophisticated managed entry agreements and spread payment approaches. For instance, although in Europe pharmaceutical pricing and reimbursement are a responsibility of each Member State, these characteristics seem to support the application of pan-European approaches to market access of advanced therapies. Specifically, there is a need for early dialogue schemes at European level and for consensus building between regulators, payers and health technology assessment agencies to align on evidence requirements (Qiu et al., 2020; Ronco et al., 2021).

Furthermore, there is a role for voluntary collaborations involving multiple countries to consider advanced therapies and jointly perform some or all of the following activities related to their market access and reimbursement: horizon scanning, health technology assessment, price negotiation, procurement, information sharing. For instance, FiNoSe has conducted a joint health technology assessment of Zynteglo® (FiNoSe, 2019) in 2019 and BeNeLuxA is carrying out a joint health technology assessment of Zolgensma® (as of April 2021) (BeNeLuxA, 2020). By pooling resources and expertise across countries, such collaborations have been shown to offer flexible pathways to design innovative approaches to value assessment, managed entry and payment for advanced therapies and other medicines (Fernandes and Kumar, 2021).

## Conclusions and Recommendations

This manuscript has argued and documented that there is no single approach to market access and reimbursement of advanced therapies. Policy and decision makers need to be aware of the varied landscape of advanced therapies and their indications, and we propose that they implement diverse, yet coherent health economic assessment frameworks and market access instruments that fit characteristics of these products and their local decision making context. The system should not be overhauled following the introduction of advanced therapies, but can be further developed to be able to adopt such innovative products. Therefore, the following recommendations do not only apply to advanced therapies, but may also be relevant for other types of innovative medicines:

• Recommendations for economic evaluation:
  • Stakeholders need to appreciate that high acquisition costs of advanced therapies can be offset by long-term health care savings and health gains. Hence, stakeholders need to consider not only the budget impact and affordability of advanced therapies, but also their cost-effectiveness as investigated in economic evaluations.
  • In addition to their traditional perspective, we advise payers to consider a scenario which encompasses all value elements that are relevant to advanced therapies.
  • There is scope for health economists to explore novel methodological approaches which allow to address the limitations of clinical evidence about advanced therapies for the purpose of their economic evaluation and reimbursement. In particular, guidance is required on how to use evidence from single-arm trials and how to extrapolate survival data.

• Managed access and policy recommendations:
  • Manufacturers and payers should not assume that spread payments are required to make advanced therapies affordable, but need to identify criteria (such as disease incidence and prevalence) on the basis of which they can decide whether a single payment or spread payments to fund acquisition costs of advanced therapies are most suitable.
  • Manufacturers and payers would benefit from developing a decision tree to guide the choice for a particular type of financial-based or outcome-based managed entry agreement to address specific (budgetary and clinical) uncertainties associated with individual advanced therapies.
  • We suggest that stakeholders develop a roadmap providing practical guidance on how outcome-based managed entry agreements for advanced therapies can be designed and rolled out in a specific jurisdiction in terms of data collection, outcome selection, payment correction, funding, IT infrastructure and data ownership.
  • Policy makers can consider strengthening multinational collaborations on access to medicines and further invest in their activities related to horizon scanning, health technology assessment, price negotiation, procurement and information sharing.

## Data Availability

The original contributions presented in the study are included in the article/Supplementary Material, further inquiries can be directed to the corresponding author.

## References

[B1] Aballéa S. Thokagevistk K. Velikanova R. Simoens S. Annemans L. Antonanzas F. (2020). Health Economic Evaluation of Gene Replacement Therapies: Methodological Issues and Recommendations. J. Mark Access Health Pol. 8 (1), 1822666. 10.1080/20016689.2020.1822666 PMC7580851 33144927

[B2] Agenzia Italiana del Farmaco (2021). Registri Farmaci Sottoposti a Monitoraggio [Online]. Available at: https://www.aifa.gov.it/web/guest/registri-farmaci-sottoposti-a-monitoraggio (Accessed).

[B3] Ali F. (2020). The Heart of Market Access: Opportunities and Challenges for Cell and Gene Therapy Development for Orphan and Prevalent Cardiovascular Diseases. Cell Gene Ther. Insights 6 (7), 1141–1152. 10.18609/cgti.2020.124

[B4] Alliance for Regenerative Medicine (2021). 2020: Growth & Resilience in Regenerative Medicine. Annual Report [Online]. Available at: http://alliancerm.org/wp-content/uploads/2021/03/ARM_AR2020_FINAL-PDF.pdf (Accessed).

[B5] American Society of Gene and Cell Therapy (2020). COVID-19 Vaccine Candidates Show Gene Therapy Is a Viable Strategy [Online]. Available at: https://www.asgct.org/research/news/november-2020/covid-19-moderna-nih-vaccine (Accessed).

[B6] Angelis A. Naci H. Hackshaw A. (2020). Recalibrating Health Technology Assessment Methods for Cell and Gene Therapies. Pharmacoeconomics 38 (12), 1297–1308. 10.1007/s40273-020-00956-w 32960434

[B7] Annemans L. (2019). A Proposal for Value Informed, Affordable ("via") Prices for Innovative Medicines. J. Med. Econ. 22 (11), 1235–1239. 10.1080/13696998.2019.1632203 31199174

[B8] Annemans L. Beutels P. Bloom D. E. De Backer W. Ethgen O. Luyten J. (2021). Economic Evaluation of Vaccines: Belgian Reflections on the Need for a Broader Perspective. Value Health 24 (1), 105–111. 10.1016/j.jval.2020.09.005 33431141

[B9] APM Health Europe (2019). Major German Payers Sign Pay for Performance Agreements on CAR-Ts [Online]. Available at: https://www.apmhealtheurope.com/freestory/0/64434/major-german-payers-sign-pay-for-performance-agreements-on-car-ts (Accessed).

[B10] Armoiry X. Cummins E. Connock M. Metcalfe A. Royle P. Johnston R. (2019). Autologous Chondrocyte Implantation with Chondrosphere for Treating Articular Cartilage Defects in the Knee: An Evidence Review Group Perspective of a NICE Single Technology Appraisal. Pharmacoeconomics 37 (7), 879–886. 10.1007/s40273-018-0737-z 30426462

[B11] Baumgardner J. R. Brauer M. S. Zhang J. Hao Y. Liu Z. Lakdawalla D. N. (2020). CAR-T Therapy and Historical Trends in Effectiveness and Cost-Effectiveness of Oncology Treatments. J. Comp. Eff. Res. 9 (5), 327–340. 10.2217/cer-2019-0065 32056442

[B12] BeNeLuxA (2020). Joint HTA Assessment of Zolgensma [Online]. Available at: https://beneluxa.org/news3 (Accessed).

[B13] Canadian Agency for Drugs and Technologies in Health (2018). CADTH Review Process for Cell and Gene Therapies [Online]. Available at: https://www.cadth.ca/sites/default/files/cdr/process/CADTH_Gene_Process.pdf (Accessed).

[B14] CEPS LEEM (2021). Accord-cadre du 05/03/2021 entre le Comité Economique des Produits de Santé et les Entreprises du Médicament [Online]. Available at: https://solidarites-sante.gouv.fr/IMG/pdf/accord_cadre_21-24_signe.pdf (Accessed).

[B15] Chambers J. Silver M. C. Lin P. J. Cohen J. T. Paramore C. Baumann S. (2019). Pmu77 Cell and Gene Therapies Are Associated with Substantially Larger Quality-Adjusted Life Year Gains Than Conventional Drugs and Biologics. Value in Health 22 (S2), S263. 10.1016/j.jval.2019.04.1238

[B16] Cher B. P. Gan K. Y. Aziz M. I. A. Lin L. Hwang W. Y. K. Poon L. M. (2020). Cost Utility Analysis of Tisagenlecleucel vs Salvage Chemotherapy in the Treatment of Relapsed/refractory Diffuse Large B-Cell Lymphoma from Singapore's Healthcare System Perspective. J. Med. Econ. 23 (11), 1321–1329. 10.1080/13696998.2020.1808981 32780608

[B17] ClinicalTrials.gov (2010). Provenge® (Sipuleucel-T) Active Cellular Immunotherapy Treatment of Metastatic Prostate Cancer after Failing Hormone Therapy [Online]. Available at: https://clinicaltrials.gov/ct2/show/study/NCT00065442 (Accessed).

[B18] Colasante W. (2019). What Risks Do Annuity Pricing Models Present to Cell & Gene Therapy Developers? [Online]. Available at: https://www.cellandgene.com/doc/what-risks-do-annuity-pricing-models-present-to-cell-gene-therapy-developers-0001 (Accessed).

[B19] Connock M. Andronis L. Auguste P. Dussart C. Armoiry X. (2020). Will the US$5 Million Onasemnogene Abeparvosec Treatment for Spinal Muscular Atrophy Represent 'value for Money' for the NHS? A Rapid Inquiry into Suggestions that it May Be Cost-Effective. Expert Opin. Biol. Ther. 20 (7), 823–827. 10.1080/14712598.2020.1772747 32434404

[B20] Cook K. Forbes S. P. Adamski K. Ma J. J. Chawla A. Garrison L. P. Jr. (2020). Assessing the Potential Cost-Effectiveness of a Gene Therapy for the Treatment of Hemophilia A. J. Med. Econ. 23 (5), 501–512. 10.1080/13696998.2020.1721508 31971453

[B21] Coyle D. Durand-Zaleski I. Farrington J. Garrison L. Graf von der Schulenburg J. M. Greiner W. (2020). HTA Methodology and Value Frameworks for Evaluation and Policy Making for Cell and Gene Therapies. Eur. J. Health Econ. 21 (9), 1421–1437. 10.1007/s10198-020-01212-w 32794011

[B22] Dabbous M. Chachoua L. Caban A. Toumi M. (2020). Managed Entry Agreements: Policy Analysis from the European Perspective. Value Health 23 (4), 425–433. 10.1016/j.jval.2019.12.008 32327159

[B23] Dabbous M. Toumi M. Simoens S. Wasem J. Saal G. Wang Y. (2022). Amortization of Gene Replacement Therapies: A Health Policy Analysis Exploring a Mechanism for Mitigating Budget Impact of High-Cost Treatments. Health Policy 126, 49–59. 10.1016/j.healthpol.2021.11.005 34863529

[B24] Drummond M. F. Neumann P. J. Sullivan S. D. Fricke F. U. Tunis S. Dabbous O. (2019). Analytic Considerations in Applying a General Economic Evaluation Reference Case to Gene Therapy. Value Health 22 (6), 661–668. 10.1016/j.jval.2019.03.012 31198183

[B25] EHDEN (2022). European Health Data & Evidence Network [Online]. Available at: https://www.ehden.eu (Accessed).

[B26] European Commission (2007). Regulation (EC) No 1394/2007 of the European Parliament and of the Council of 13 November 2007 on Advanced Therapy Medicinal Products and Amending Directive 2001/83/EC and Regulation (EC) No 726/2004. Official Journal of the European Union. Available at: https://ec.europa.eu/health//sites/health/files/files/eudralex/vol-1/reg_2007_1394/reg_2007_1394_en.pdf . L 324/121.

[B27] Eurostat (2013). European System of Accounts - ESA 2010 [Online]. Available at: https://ec.europa.eu/eurostat/web/products-manuals-and-guidelines/-/KS-02-13-269 (Accessed).

[B28] Faulkner E. Spinner D. S. Ringo M. Carroll M. (2019). Are Global Health Systems Ready for Transformative Therapies? Value Health 22 (6), 627–641. 10.1016/j.jval.2019.04.1911 31198179

[B29] Fernandes J. Kumar A. (2021). BeNeLuxA & FiNoSe Joint Initiative: Are They as Beneficial to Manufacturers as They Seem? [Online]. Available at: https://partners4access.com/beneluxa-and-finose-joint-initiative/ (Accessed).

[B30] FiNoSe (2019). Zynteglo (Autologous Cd34+ Cells Encoding βA-T87Q-globin Gene). FINOSE Joint Assessment Report [Online]. Available at: https://www.tlv.se/download/18.1ee533eb171f50617c136774/1589207033570/bes200430_zynteglo_english.pdf (Accessed).

[B31] Flume M. Bardou M. Capri S. Sola-Morales O. Cunningham D. Levin L. A. (2018). Approaches to Manage 'affordability' of High Budget Impact Medicines in Key EU Countries. J. Mark Access Health Pol. 6 (1), 1478539. 10.1080/20016689.2018.1478539 PMC5998770 29915664

[B32] Foxon G. Craddy P. Walker L. (2019). Are Acute Therapies and Curative Drugs More Affordable Than Chronic Treatments in Rare Diseases? an Analysis of the Top 20 Most Expensive Drugs in the US [Online]. Available at: https://www.remapconsulting.com/wp-content/uploads/2019/10/ISPOR-2019-High-cost-drugs-poster-0_1.pdf (Accessed).

[B33] Furzer J. Gupta S. Nathan P. C. Schechter T. Pole J. D. Krueger J. (2020). Cost-effectiveness of Tisagenlecleucel vs Standard Care in High-Risk Relapsed Pediatric Acute Lymphoblastic Leukemia in Canada. JAMA Oncol. 6 (3), 393–401. 10.1001/jamaoncol.2019.5909 31971547 PMC6990832

[B34] GWQ Service Plus (2020). Agreement between AveXis and GWQ for the Performance-Oriented Reimbursement of Zolgensma® [Online]. Available at: https://www.gwq-serviceplus.de/aktuelles/news/vereinbarung-gwq-p4p-vertrag-zu-zolgensma_6050 (Accessed).

[B35] GWQ Service Plus (2019). Novartis Pharma GmbH und GWQ ServicePlus AG schließen Vertrag über ein innovatives Erstattungsmodell für die CAR-T-Zelltherapie [Online]. Available at: https://www.gwq-serviceplus.de/aktuelles/news/novartis-gwq-car-t-zelltherapie-06-03-2019_5178 (Accessed).

[B36] Autorité de Santé H. (2020). Zynteglo: Avis D'efficience [Online]. Available at: https://www.has-sante.fr/upload/docs/application/pdf/2020-05/zynteglo_11022020_avis_efficience.pdf (Accessed).

[B37] Hao Y. Eldjerou L. K. Yang H. Qi C. Globe D. (2017). Cost-effectiveness Analysis of CTL019 for the Treatment of Pediatric and Young Adult Patients with Relapsed or Refractory B-Cell Acute Lymphoblastic Leukemia in the United States. Blood 130 (Suppl. 1), 609. 10.1182/blood.V130.Suppl_1.609.609

[B38] Healthcare Improvement Scotland (2019). Guidance to Submitting Companies for Completion of New Product Assessment Form (NPAF): Supplement for Medicines for Extremely Rare Conditions (Ultra-orphan Medicines) [Online]. Available at: https://www.scottishmedicines.org.uk/media/5801/guidance-supplement-ultra-orphan-updated-011119.pdf (Accessed).

[B39] Hettle R. Corbett M. Hinde S. Hodgson R. Jones-Diette J. Woolacott N. (2017). The Assessment and Appraisal of Regenerative Medicines and Cell Therapy Products: an Exploration of Methods for Review, Economic Evaluation and Appraisal. Health Technol. Assess. 21 (7), 1–204. 10.3310/hta21070 PMC5337841 28244858

[B40] Horgan D. Metspalu A. Ouillade M. C. Athanasiou D. Pasi J. Adjali O. (2020). Propelling Healthcare with Advanced Therapy Medicinal Products: A Policy Discussion. Biomed. Hub 5 (3), 130–152. 10.1159/000511678 33987187 PMC8101061

[B41] Institute for Clinical and Economic Review (2019a). Adapted Value Assessment Methods for High-Impact “Single and Short-Term Therapies” (SSTs) [Online]. Available at: https://icer.org/wp-content/uploads/2020/10/ICER_SST_FinalAdaptations_111219.pdf (Accessed).

[B42] Institute for Clinical and Economic Review (2019b). Spinraza® and Zolgensma® for Spinal Muscular Atrophy: Effectiveness and Value [Online]. Available at: https://icer.org/wp-content/uploads/2020/10/ICER_SMA_Final_Evidence_Report_110220.pdf (Accessed).

[B43] Jansen J. P. Fleurence R. Devine B. Itzler R. Barrett A. Hawkins N. (2011). Interpreting Indirect Treatment Comparisons and Network Meta-Analysis for Health-Care Decision Making: Report of the ISPOR Task Force on Indirect Treatment Comparisons Good Research Practices: Part 1. Value Health 14 (4), 417–428. 10.1016/j.jval.2011.04.002 21669366

[B44] Johnson S. Buessing M. O'Connell T. Pitluck S. Ciulla T. A. (2019). Cost-effectiveness of Voretigene Neparvovec-Rzyl vs Standard Care for RPE65-Mediated Inherited Retinal Disease. JAMA Ophthalmol. 137 (10), 1115–1123. 10.1001/jamaophthalmol.2019.2512 31318398 PMC6646972

[B45] Jönsson B. Hampson G. Michaels J. Towse A. von der Schulenburg J. G. Wong O. (2019). Advanced Therapy Medicinal Products and Health Technology Assessment Principles and Practices for Value-Based and Sustainable Healthcare. Eur. J. Health Econ. 20 (3), 427–438. 10.1007/s10198-018-1007-x 30229376 PMC6438935

[B46] Jørgensen J. Hanna E. Kefalas P. (2020). Outcomes-based Reimbursement for Gene Therapies in Practice: the Experience of Recently Launched CAR-T Cell Therapies in Major European Countries. J. Mark Access Health Pol. 8 (1), 1715536. 10.1080/20016689.2020.1715536 PMC7006635 32082514

[B47] Jørgensen J. Kefalas P. (2021). The Use of Innovative Payment Mechanisms for Gene Therapies in Europe and the USA. Regenerative Med. 16, 405–422. 10.2217/rme-2020-0169 33847163

[B49] Lakdawalla D. N. Doshi J. A. Garrison L. P. Jr. Phelps C. E. Basu A. Danzon P. M. (2018). Defining Elements of Value in Health Care-A Health Economics Approach: An ISPOR Special Task Force Report [3]. Value Health 21 (2), 131–139. 10.1016/j.jval.2017.12.007 29477390

[B50] LaMattina J. (2019). Outcomes-based Pricing Not A Panacea for High Priced Drugs [Online]. Available at: https://www.forbes.com/sites/johnlamattina/2019/03/27/outcomes-based-pricing-not-a-panacea-for-high-priced-drugs/?sh=769ad1095c23 (Accessed).

[B51] Leech A. A. Dusetzina S. B. (2019). Cost-Effective but Unaffordable: The CAR-T Conundrum. J. Natl. Cancer Inst. 111 (7), 644–645. 10.1093/jnci/djy195 30561705

[B52] Lin J. K. Lerman B. J. Barnes J. I. Boursiquot B. C. Tan Y. J. Robinson A. Q. L. (2018). Cost Effectiveness of Chimeric Antigen Receptor T-Cell Therapy in Relapsed or Refractory Pediatric B-Cell Acute Lymphoblastic Leukemia. J. Clin. Oncol. 36 (32), 3192–3202. 10.1200/JCO.2018.79.0642 30212291

[B53] Lin J. K. Muffly L. S. Spinner M. A. Barnes J. I. Owens D. K. Goldhaber-Fiebert J. D. (2019). Cost Effectiveness of Chimeric Antigen Receptor T-Cell Therapy in Multiply Relapsed or Refractory Adult Large B-Cell Lymphoma. J. Clin. Oncol. 37 (24), 2105–2119. 10.1200/JCO.18.02079 31157579

[B54] Maes I. Boufraioua H. Schoonaert L. Van Dyck W. (2019). Innovative Solutions for Paradigm Changing New Therapies – Policy Report Based on Multi-Stakeholder Round Tables [Online]. Available at: https://www.inovigate.com/media/filer_public/e8/9c/e89ca2b0-1dcf-48fb-9afc-9e911ddcef84/innovative_funding_solutions_-_short_version_without_appendix_vs09.pdf (Accessed).

[B55] Malone D. C. Dean R. Arjunji R. Jensen I. Cyr P. Miller B. (2019). Cost-effectiveness Analysis of Using Onasemnogene Abeparvocec (AVXS-101) in Spinal Muscular Atrophy Type 1 Patients. J. Mark Access Health Pol. 7 (1), 1601484. 10.1080/20016689.2019.1601484 PMC6508058 31105909

[B56] Marchetti M. Martelli E. Zinzani P. L. (2018). Cost-effectiveness of Axicabtagene Ciloleucel for Relapsed or Refractory Diffuse Large B-Cell Lymphoma in Italy. Blood 132 (Suppl. 1), 4779. 10.1182/blood-2018-99-113838

[B57] McConaghie A. (2019). Europe Responding to Gene Therapy challenge, but Picture Remains Fragmented [Online]. Available at: https://www.pmlive.com/pharma_news/europe_responding_to_gene_therapy_challenge,_but_picture_remains_fragmented_1288772 (Accessed).

[B58] Michelsen S. Nachi S. Van Dyck W. Simoens S. Huys I. (2020). Barriers and Opportunities for Implementation of Outcome-Based Spread Payments for High-Cost, One-Shot Curative Therapies. Front. Pharmacol. 11, 594446. 10.3389/fphar.2020.594446 33363468 PMC7753155

[B59] Ministerio de Sanidad, Consumo y Bienestar Social (2020). Acuerdos de la reunión de la comisión interministerial de precios de los medicamentos. Sesión 208 de 17 de diciembre de 2020 [Online]. Available at: https://www.mscbs.gob.es/profesionales/farmacia/pdf/Acuerdos_CIPM_208_de_17_de_DICIEMBRE_web.pdf (Accessed).

[B60] Ministerio de Sanidad, Consumo y Bienestar Social (2019). Sistema de Información para determinar el Valor Terapéutico en la Práctica Clínica Real de los Medicamentos de Alto Impacto Sanitario y Económico en el SNS (VALTERMED) [Online]. Available at: https://www.mscbs.gob.es/profesionales/farmacia/valtermed/home.htm (Accessed).

[B61] National Institute for Health and Care Excellence (2019a). Axicabtagene Ciloleucel for Treating Diffuse Large B-Cell Lymphoma and Primary Mediastinal Large B-Cell Lymphoma after 2 or More Systemic Therapies. Technology Appraisal Guidance [Online]. Available at: https://www.nice.org.uk/guidance/ta559/resources/axicabtagene-ciloleucel-for-treating-diffuse-large-bcell-lymphoma-and-primary-mediastinal-large-bcell-lymphoma-after-2-or-more-systemic-therapies-pdf-82607030270917 (Accessed). 40080619

[B62] National Institute for Health and Care Excellence (2017). Budget Impact Test [Online]. Available at: https://www.nice.org.uk/about/what-we-do/our-programmes/nice-guidance/nice-technology-appraisal-guidance/budget-impact-test (Accessed).

[B63] National Institute for Health and Care Excellence (2019b). Cancer Drugs Fund Managed Access Agreement Tisagenlecleucel for Treating Relapsed or Refractory Diffuse Large B-Cell Lymphoma after 2 or More Systemic Therapies [TA567] [Online]. Available at: https://www.nice.org.uk/guidance/ta567/resources/managed-access-agreement-march-2019-pdf-6718513213 (Accessed). 40228081

[B64] National Institute for Health and Care Excellence (2021a). Commercial Access Agreement: KTE-X19 for Treating Relapsed or Refractory Mantle Cell Lymphoma [ID1313] [Online]. Available at: https://www.nice.org.uk/guidance/ta677/documents/final-appraisal-determination-document (Accessed).

[B65] National Institute for Health and Care Excellence (2021b). Final Appraisal Document. Autologous Anti-CD19-transduced CD3+ Cells for Treating Relapsed or Refractory Mantle Cell Lymphoma [Online]. Available at: https://www.nice.org.uk/guidance/ta677/documents/final-appraisal-determination-document (Accessed).

[B66] National Institute for Health and Care Excellence (2022). Review of Methods, Processes and Topic Selection for Health Technology Evaluation Programmes: Conclusions and Final Update. Appendix: Further Discussion and Rationale for Conclusions – Methods [Online]. Available at: https://view.officeapps.live.com/op/view.aspx?src=https%3A%2F%2Fwww.nice.org.uk%2FMedia%2FDefault%2FAbout%2Fwhat-we-do%2Four-programmes%2Ftechnology-appraisals%2Fmethods-processes-and-topic-selection-review-board-paper-appendix.docx&wdOrigin=BROWSELINK (Accessed).

[B67] National Institute for Health and Care Excellence (2019c). Talimogene Laherparepvec for Treating Unresectable Metastatic Melanoma: Technology Appraisal Guidance [Online]. Available at: https://www.nice.org.uk/guidance/ta410 (Accessed).

[B69] National Institute for Health and Care Excellence (2019d). Voretigene Neparvovec for Treating Inherited Retinal Dystrophies Caused by RPE65 Gene Mutations. Highly Specialised Technologies Guidance [Online]. Available at: https://www.nice.org.uk/guidance/hst11/resources/voretigene-neparvovec-for-treating-inherited-retinal-dystrophies-caused-by-rpe65-gene-mutations-pdf-50216253809605 (Accessed). 40892954

[B70] National Institute for Health and Disability Insurance (2019). Search Engine Reimbursable Medicines [Online]. Available at: https://ondpanon.riziv.fgov.be/SSPWebApplicationPublic/nl/Public/ProductSearch (Accessed).

[B71] Nestler-Parr S. Korchagina D. Toumi M. Pashos C. L. Blanchette C. Molsen E. (2018). Challenges in Research and Health Technology Assessment of Rare Disease Technologies: Report of the ISPOR Rare Disease Special Interest Group. Value Health 21 (5), 493–500. 10.1016/j.jval.2018.03.004 29753344

[B72] NHS England (2021). Cancer Drugs Fund [Online]. Available at: https://www.england.nhs.uk/cancer/cdf/ (Accessed).

[B73] Orkin S. H. Reilly P. (2016). Paying for Future success in Gene Therapy. Science 352 (6289), 1059–1061. 10.1126/science.aaf4770 27230368

[B74] Parsons L. (2020). Bluebird Bio Launches Beta Thalassaemia Gene Therapy Zynteglo in Germany [Online]. Available at: http://www.pmlive.com/pharma_news/bluebird_bio_launches_beta_thalassaemia_gene_therapy_zynteglo_in_germany_1322226 (Accessed).

[B75] Partners4Access (2019). Evolution of Payment Models for Cell and Gene Therapies in Italy [Online]. Available at: https://partners4access.com/evolution-of-payment-models-for-cell-and-gene-therapies-in-italy/ (Accessed).

[B76] Pearson S. D. (2018). The ICER Value Framework: Integrating Cost Effectiveness and Affordability in the Assessment of Health Care Value. Value Health 21 (3), 258–265. 10.1016/j.jval.2017.12.017 29566831

[B77] Qiu T. Dabbous M. Borissov B. (2020). The Evaluation of Pivotal Trials for Advanced Therapies from Regulatory and Health Technology Assessment Perspectives in Europe. Value & Outcomes Spotlight 6, 33–36.

[B78] Remap Consulting (2019). Gene Therapies and Managed Entry Agreements. How Willing Have Payers Been to Cover High Cost Therapies Such as Zolgensma and Zynteglo? [Online]. Available at: https://remapconsulting.com/gene-therapies-and-managed-entry-agreements-how-willing-have-payers-been-to-cover-high-cost-therapies-such-as-zolgensma-and-zynteglo/ (Accessed).

[B79] Ribera Santasusana J. M. de Andrés Saldaña A. García-Muñoz N. Gostkorzewicz J. Martínez Llinàs D. Díaz de Heredia C. (2020). Cost-Effectiveness Analysis of Tisagenlecleucel in the Treatment of Relapsed or Refractory B-Cell Acute Lymphoblastic Leukaemia in Children and Young Adults in Spain. Clinicoecon Outcomes Res. 12, 253–264. 10.2147/CEOR.S241880 32523362 PMC7237114

[B80] Ridderstad Wollberg A. (2020). Final Report for Swelife - ATMP System Development Project 3 (SDP 3) [Online]. Available at: https://atmpsweden.se/wp-content/uploads/2021/03/Final-rapport-dec-2020_final_ENG_v3.pdf (Accessed).

[B81] Ronco V. Dilecce M. Lanati E. Canonico P. L. Jommi C. (2021). Price and Reimbursement of Advanced Therapeutic Medicinal Products in Europe: Are Assessment and Appraisal Diverging from Expert Recommendations? J. Pharm. Pol. Pract 14 (1), 30. 10.1186/s40545-021-00311-0 PMC7980570 33741076

[B82] Rosa F. (2019). Hemos co-creado un modelo con el Ministerio para introducir el CAR-T [Online]. Available at: https://www.diariofarma.com/2019/04/23/hemos-co-creado-un-modelo-con-el-ministerio-para-introducir-el-car-t (Accessed).

[B83] Roth J. A. Sullivan S. D. Lin V. W. Bansal A. Purdum A. G. Navale L. (2018). Cost-effectiveness of Axicabtagene Ciloleucel for Adult Patients with Relapsed or Refractory Large B-Cell Lymphoma in the United States. J. Med. Econ. 21 (12), 1238–1245. 10.1080/13696998.2018.1529674 30260711

[B84] Sarkar R. R. Gloude N. J. Schiff D. Murphy J. D. (2019). Cost-Effectiveness of Chimeric Antigen Receptor T-Cell Therapy in Pediatric Relapsed/Refractory B-Cell Acute Lymphoblastic Leukemia. J. Natl. Cancer Inst. 111 (7), 719–726. 10.1093/jnci/djy193 30551196 PMC6624167

[B85] Schaffer S. K. Messner D. Mestre-Ferrandiz J. Tambor E. Towse A. (2018). Paying for Cures: Perspectives on Solutions to the "Affordability Issue". Value Health 21 (3), 276–279. 10.1016/j.jval.2017.12.013 29566833

[B86] Scottish Medicines Consortium (2012). SMC Modifiers Used in Appraising New Medicines [Online]. Available at: https://www.scottishmedicines.org.uk/media/3565/modifiers.pdf (Accessed).

[B87] Sheppard C. Akbraian E. Bernardini A. Wang R. (2021). Innovative Access Agreements for ATMPs in Europe: Why Embrace Them? [Online]. Partners4Access. Available at: https://partners4access.com/innovative-access-agreements-for-atmps-in-europe/ (Accessed).

[B88] Simoens S. Spriet I. (2020). Guidance for Demonstrating the Societal Value of New Antibiotics. Front. Pharmacol. 11, 618238. 10.3389/fphar.2020.618238 33597882 PMC7882732

[B89] South E. Cox E. Meader N. Woolacott N. Griffin S. (2019). Strimvelis® for Treating Severe Combined Immunodeficiency Caused by Adenosine Deaminase Deficiency: An Evidence Review Group Perspective of a NICE Highly Specialised Technology Evaluation. Pharmacoecon Open 3 (2), 151–161. 10.1007/s41669-018-0102-3 30334168 PMC6533345

[B90] Svensson M. Nilsson F. O. Arnberg K. (2015). Reimbursement Decisions for Pharmaceuticals in Sweden: The Impact of Disease Severity and Cost Effectiveness. Pharmacoeconomics 33 (11), 1229–1236. 10.1007/s40273-015-0307-6 26093889

[B91] Ten Ham R. M. T. Klungel O. H. Leufkens H. G. M. Frederix G. W. J. (2020). A Review of Methodological Considerations for Economic Evaluations of Gene Therapies and Their Application in Literature. Value Health 23 (9), 1268–1280. 10.1016/j.jval.2020.04.1833 32940245

[B92] Thielen F. W. van Dongen-Leunis A. Arons A. M. M. Ladestein J. R. Hoogerbrugge P. M. Uyl-de Groot C. A. (2020). Cost-effectiveness of Anti-CD19 Chimeric Antigen Receptor T-Cell Therapy in Pediatric Relapsed/refractory B-Cell Acute Lymphoblastic Leukemia. A Societal View. Eur. J. Haematol. 105 (2), 203–215. 10.1111/ejh.13427 32289184 PMC7497258

[B93] Towse A. Mauskopf J. A. (2018). Affordability of New Technologies: The Next Frontier. Value Health 21 (3), 249–251. 10.1016/j.jval.2018.01.011 29566829

[B94] Van Dyck W. Michelsen S. Veredas D. Huys I. Simoens S. Luyten J. (2022). Annuity-Based Payment as a Solution for “Cost-Effective but Unaffordable” Curative Therapies: Insights from a Budget Impact Model. Manuscript in preparation.

[B95] van Overbeeke E. Michelsen S. Toumi M. Stevens H. Trusheim M. Huys I. (2021). Market Access of Gene Therapies across Europe, USA, and Canada: Challenges, Trends, and Solutions. Drug Discov. Today 26 (2), 399–415. 10.1016/j.drudis.2020.11.024 33242695

[B96] Viriato D. Bennett N. Sidhu R. Hancock E. Lomax H. Trueman D. (2020). An Economic Evaluation of Voretigene Neparvovec for the Treatment of Biallelic RPE65-Mediated Inherited Retinal Dystrophies in the UK. Adv. Ther. 37 (3), 1233–1247. 10.1007/s12325-020-01243-y 32034665 PMC7089725

[B97] Wakase S. Teshima T. Zhang J. Ma Q. Watanabe Y. Yang H. (2021). Cost-Effectiveness Analysis of Tisagenlecleucel for the Treatment of Pediatric and Young Adult Patients with Relapsed or Refractory B Cell Acute Lymphoblastic Leukemia in Japan. Transplant. Cell Ther. 27 (3), 241.e1–241.e11. 10.1016/j.jtct.2020.12.023 33781519

[B98] Walton M. Sharif S. Simmonds M. Claxton L. Hodgson R. (2019). Tisagenlecleucel for the Treatment of Relapsed or Refractory B-Cell Acute Lymphoblastic Leukaemia in People Aged up to 25 years: An Evidence Review Group Perspective of a NICE Single Technology Appraisal. Pharmacoeconomics 37 (10), 1209–1217. 10.1007/s40273-019-00799-0 30982165

[B99] Whittington M. D. McQueen R. B. Ollendorf D. A. Kumar V. M. Chapman R. H. Tice J. A. (2019). Long-term Survival and Cost-Effectiveness Associated with Axicabtagene Ciloleucel vs Chemotherapy for Treatment of B-Cell Lymphoma. JAMA Netw. Open 2 (2), e190035. 10.1001/jamanetworkopen.2019.0035 30794298 PMC6484589

[B100] Whittington M. D. McQueen R. B. Ollendorf D. A. Kumar V. M. Chapman R. H. Tice J. A. (2018). Long-term Survival and Value of Chimeric Antigen Receptor T-Cell Therapy for Pediatric Patients with Relapsed or Refractory Leukemia. JAMA Pediatr. 172 (12), 1161–1168. 10.1001/jamapediatrics.2018.2530 30304407 PMC6583018

[B101] WHO Collaborating Centre for Pharmaceutical Pricing and Reimbursement Policies (2018). PPRI Pharma Profile: Norway [Online]. Available at: https://ppri.goeg.at/sites/ppri.goeg.at/files/inline-files/PPRI%20Norway%202018.pdf (Accessed).

[B102] Zimmermann M. Lubinga S. J. Banken R. Rind D. Cramer G. Synnott P. G. (2019). Cost Utility of Voretigene Neparvovec for Biallelic RPE65-Mediated Inherited Retinal Disease. Value Health 22 (2), 161–167. 10.1016/j.jval.2018.09.2841 30711060

[B103] Zorginstituut Nederland (2021). Sluis Voor Dure Geneesmiddelen [Online]. Available at: https://www.zorginstituutnederland.nl/over-ons/programmas-en-samenwerkingsverbanden/horizonscan-geneesmiddelen/sluis-voor-dure-geneesmiddelen (Accessed).

[B104] Zwaap J. Knies S. van der Meijden C. Staal P. van der Heiden L. (2015). Kosteneffectiviteit in de praktijk [Online]. Available at: https://www.zorginstituutnederland.nl/publicaties/rapport/2015/06/26/kosteneffectiviteit-in-de-praktijk (Accessed).

